# Medical student selection criteria and junior doctor workplace performance

**DOI:** 10.1186/s12909-019-1829-y

**Published:** 2019-10-21

**Authors:** Ruth M. Sladek, Christine Burdeniuk, Alison Jones, Kevin Forsyth, Malcolm J. Bond

**Affiliations:** 1 Prideaux Centre for Research in Health Professions Education, College of Medicine and Public Health, Flinders University, GPO Box 2100, Adelaide, 5001 Australia; 2 Department of Cardiology, Southern Adelaide Local Health Network, Flinders Drive, Bedford Park, 5042 Australia; 3 College of Medicine and Public Health, Flinders University, GPO Box 2100, Adelaide, 5001 Australia

**Keywords:** Academic performance, Grade point average, Medical students, Interviews, Internship and residency, Selection tests, Student selection, Workplace performance

## Abstract

**Background:**

Medical school selection decisions have consequences beyond graduation. With generally low attrition rates, most medical students become junior doctors. Universities are therefore not just selecting students into a medical course; they are choosing the future medical workforce. Understanding the relationship between selection criteria and outcomes beyond the successful completion of a medical degree may inform approaches to student selection.

**Methods:**

A retrospective data matching study was conducted involving 39 interns employed by a South Australian local health network in 2017 who had originally entered Flinders University’s medical school through a graduate pathway. Student selection data were matched with internship workplace performance scores (measured by supervising consultants’ reports across five clinical rotations using a standardised assessment). Correlational analyses then examined associations between these two sets of variables.

**Results:**

An overall selection rank (equal thirds of weighted Grade Point Average from a prior degree, a panel interview, and a national selection test) was moderately associated with all performance measures, accounting for up to 25% of variance. Both weighted Grade Point Average and the interview had multiple and mostly moderate correlations with performance. An increasing number of years taken to complete the course was associated with poorer workplace performance across multiple outcome measures (moderate to strong negative associations with 31 to 62% of shared variance), as was age to a lesser extent (7 to 14%). The national selection test contributed a single and small relationship accounting for 5% of variance with one outcome measure.

**Conclusions:**

Selection into medicine is a critical assessment given that most students become doctors. This study found multiple associations between selection scores and junior doctor workplace performance measures in the internship year, with weighted Grade Point Average from a prior degree and an interview appearing more important than the national selection test. Future collaborative research should map desired workplace performance outcomes to initial student selection and explore the impact of changes to selection which focus on assessment of these domains. The association between slower course progression and poorer workplace performance should also be examined.

## Background

Medical school selection decisions may have long term consequences post-graduation. With generally low attrition rates [1–3] most medical students become junior doctors. Universities are not just selecting students into a medical course; they are choosing the future medical workforce. Understanding the relationship between selection criteria and outcomes beyond the successful completion of a medical degree may inform approaches to student selection. This paper reports an exploratory study examining associations between student selection criteria and junior doctor workplace performance.

Selection into medicine uses a range of tools that generally assess cognitive and personal qualities [4], embracing measures such as prior academic achievement, aptitude tests, personal statements and/or curriculum vitae, references and letters of recommendation, situational judgement tests (SJTs), personality assessment, and a variety of interview formats [5]. Not only are different tools used by different medical programs, but there are differences in the ways in which scores are combined to select students. Despite these variations the initial endpoint post-graduation is the same: entry into the medical workforce. Yet selection research has focused on in-course performance only rather than longitudinal outcomes [6]. When data do extend past in-course results, the focus is usually on ‘achievement’ (e.g., scores on licensing examinations) rather than day to day workplace performance (e.g., usual behaviours in clinical practice) [5, 6]. These are conceptually different, reflecting what doctors *can* do compared with what they *actually* do [7]. A patient (and an employer) may want a doctor who performs well in a controlled, high stakes assessment of competence such as the United States Medical Licencing Examinations (USMLEs), but certainly also wants a doctor who performs well in the actual care they deliver.

Currently little is known about relationships between student selection and workplace performance [6], particularly for junior doctors [8]. Studies examining Medical Course Admissions Test (MCAT) scores, perhaps the most well-known selection test, in relation to workplace performance have failed to find meaningful associations. For example, MCAT was unrelated to program directors’ evaluation of residents’ performance [9], “knowledge and clinical capabilities”, and “professionalism” rating scales [10]. Perhaps this is unsurprising. Selection criteria that focus on cognitive or knowledge domains might conceivably be associated with longer term measures of achievement, but they are not generally designed to assess an individual’s likely workplace performance.

Conversely, selection tools that assess personal qualities (e.g., interviews) are more likely to be associated with work performance. The proposal that qualities such as communication might be associated with performance in clinical practice certainly has appealing face validity. One common approach to assessing personal qualities is the multiple mini interview (MMI). As yet the ability of MMIs to predict workplace performance has received little attention [11, 12].

Situational judgement tests (SJTs) are also used to assess personal qualities. Whilst an early systematic review found sufficient evidence to support the predictive ability of such tests for job performance [13], none of the included studies related to medicine. A subsequent review of SJTs in medical education cites only two longitudinal studies relating to the use of SJTs during student selection [14], where they were positively associated with internship performance and workplace performance 9 years later [15, 16].

Few selection criteria beyond MCAT, MMIs and SJTs have been examined. An analysis of a random sample of admissions committee member tertiary reviews found a small but significant positive association between negative interview comments and lower “professionalism” scores as assessed by program directors at the end of the internship year [17]. In the Australian context, interns who had entered medical school with a mixed background (science and humanities) performed better at internship than those with a science only background, as assessed by their clinical supervisors. Other measures, such as previous academic performance, did not produce significant effects [18]. Selection scores for entry into an undergraduate Australian medical course (Undergraduate Medical Admissions Test, secondary school academic performance, and selection interview) were not significantly correlated with workplace performance in the internship year as assessed by clinical supervisors [8].

In summary, scant research has examined workplace performance, and given that selection criteria may predict differentially across the entire medical curriculum [6], the perceived need for longitudinal and collaborative studies is unsurprising [6, 8]. Such studies are complex and costly and, before investing in such research, more preliminary data are necessary to inform research questions around the relationship between selection and performance. We contribute to this by exploring relationships between common selection criteria and junior doctor workplace performance.

### Background to this study

This study is contextualised in an Australian graduate entry medical school which is located within a major teaching health service where many, but not all, of its graduates also complete their internship. Selection of graduate entry applicants has remained stable since 1996 and includes three elements: a cognitive test (Graduate Australian Medical School Admissions Test: GAMSAT), academic performance (a weighted Grade Point Average of an undergraduate degree: wGPA), and assessment of personal qualities (panel interview).

The intern year is a transition to practice year, referred to as Postgraduate Year 1 (PGY1), which must be satisfactorily completed for a graduate to receive general registration as a medical practitioner in Australia. It comprises 47 weeks of supervised clinical experience, at the end of which the employing health agency must certify satisfactory completion of required rotations.

## Methods

### Design

A retrospective data matching method was utilised.

### Participants

‘Selection’ and ‘assessment’ data were available for 39 interns who had entered medical school via a graduate entry pathway at Flinders University and subsequently undertook their internship in the Southern Adelaide Local Health Network (SALHN) in 2017.

### Selection data

In order to be offered a medical school place, graduate applicants require three selection scores. The first is a weighted Grade Point Average (wGPA) from a prior degree at Bachelor level (in any field). Second, a valid national selection test result using the “Graduate Australian Medical Schools Admissions Test” (GAMSAT) is required. These scores can vary from 50 to 100 and encompass Reasoning in the Humanities and Social Sciences (Section 1), Written Communication (Section 2), Reasoning in the Biological Sciences (Section 3) and a Total score (which is weighted in favour of Section 3). Applicants with the highest GAMSAT Total scores are invited to an interview, which provides the third and final selection score for place offer consideration. Interview scores are expressed as a percentage and reflect the scores of 3 interviewers in a panel interview across seven domains: communication, motivation, decision-making, learning style, prosocial attitudes, personal management and a global rating. This latter domain reflects an interviewer’s overall assessment of a candidate’s suitability for the medical profession and is not an aggregate of the other six domains. This interview is highly structured, with standardised questions, a common rating scale and administration and there is pre-requisite interviewer training. It was designed to assess domains identified through public consultation as relevant to the medical school, the medical profession and society.

After their interviews, applicants are accorded an overall selection score based on a combination of their GAMSAT Total score, wGPA and Interview results, with all three components given equal weighting. Applicants are then ranked for place offer based on this selection score.

A range of other characteristics to describe interns were recorded such as age, type of prior undergraduate degree (categorised as per Craig [19]), number of years taken to complete the medical course, and rural background.

### Outcome variables

Each intern undertook rotations in 5 of 44 possible clinical areas. Nearly all interns had a mid-term *and* end of term assessment for each rotation, generating a usual maximum of 10 assessments. A standardised “Intern Training – Intern Assessment form” was used for all assessments, with outcomes defined according to a National Framework for Medical Internship, which includes “the intern as scientist and scholar” and “the intern as practitioner” [20]. There were 7 missing mid-term assessments and 6 missing end of term assessments in the current dataset. The total number of available assessments for analysis were: (48 interns × 10 assessments) – 13 missing = 467 assessments, providing an extensive dataset for analysis.

Assessments covered the following domains using a 5-point Likert scale (1 = low, 5 = high):

Domain 1: Science and Scholarship – the Intern as Scientist and Scholar (1 question); Domain 2: Clinical Practice – the Intern as Practitioner (9 questions); Domain 3: Health and Society – the Intern as a Health Advocate (4 questions); Domain 4: Professionalism and Leadership – the Intern as a Professional and Leader (6 questions).

Assessors also indicated whether an Improving Performance Action Plan (IPAP) was required (Yes/No), which is a trigger for the development of a tailored and supportive plan for the intern. A global rating of satisfactory, borderline or unsatisfactory for overall workplace performance was additionally allocated. Collectively these assessments are designed to support interns in their workplace performance.

Mean scores for Domains 1 to 4 were calculated for all available assessments for every intern, providing that a prerequisite number of questions were completed by the assessor. These were valid responses for one of one question (Domain 1, Science and Scholarship), seven of 9 questions (Domain 2, Clinical Practice), two of four questions (Domain 3, Health and Society) and five of six questions (Domain 4, Professionalism and Leadership). Mean global scores were calculated and the need for an Improving Performance Action Plan noted.

Because more than 50% of responses to Domain 3 (Health and Society) questions were omitted in assessments (as the relevant behaviours were not observed during that rotation) this Domain was excluded from subsequent analyses. Mid-term assessments were also omitted because there was a greater likelihood that the final assessments were completed by a supervising consultant (as opposed to a senior trainee). The exception was the requirement for an Improving Performance Action Plan, which was counted from mid-term assessments as it was deemed unlikely such a plan would be requested without the knowledge of the consultant. Unless stated otherwise the outcome measures listed in Table 1 were calculated from end of term assessments.

**Table 1:** Outcome Measures

Name	Definition
Domain 1 Performance	Mean Domain 1 (Science and Scholarship) In all End of Term 1–5 Intern assessments
Domain 2 Performance	Mean Domain 2 (Clinical Practice) In all End of Term 1–5 Intern assessments
Domain 4 Performance	Mean Domain 4 (Health and Society) In all End of Term 1–5 Intern assessments
Total Performance	Mean Domain 1 + Domain 2 + Domain 4 In End of Term 1–5 Intern assessments
No. of IPAPs	Total number of mid and end of term IPAPs required (Improving Performance Action Plans)
Global Performance	Mean Global Rating in End of Terms 1–5 Intern assessments based on a single assessment of performance on each placement as satisfactory, borderline or unsatisfactory

### Statistical analyses

Quantitative data were analysed using IBM SPSS Statistics Version 25.0. Assessment and selection data were first entered into two separate files, then matched using a Master List ID number, and reallocated a randomly generated ID using SPSS functionality to ensure data remained de-identified.

Multivariate statistics were not used due to the small sample size. Pearson’s Product Moment correlation coefficients explored possible linear relationships. Statistical significance for all analyses was set at *p* < 0.05 (2-tailed). However, again due to small sample size, an effect size (literally magnitude of the correlations) of at least 0.2 was determined, a priori, to be noteworthy.

## Results

### Sociodemographic data (see Table 2)

There was an equivalent gender balance, with 20/39 (51.3%) female. Only 6/39 (15.4%) had undergraduate qualifications categorised as ‘Non-Science’. A total of 12/39 (30.8%) had underlying health professional qualifications, and 14/39 (35.9%) had a biomedical sciences background. Most interns had completed their medical course in the minimum time of 4 years (28/39, 71.8%), although five had taken 5 years, three took 6 years, two took 7 years, and one took 8 years.

**Table 2:** Sociodemographic Characteristics of Total Cohort (*n* = 39)

	Graduate Entrants
Citizenship	Australian 36, New Zealand 2, International 1^a^
Gender (*n*, % female)	20, 51.3%
Age in Years (M, SD; Mdn; Range)	26.7, 5.9; 25; 19–46
Rural (*n*, %)	11, 28.2%
Undergraduate Degree	Health Professions 12 Biomedical Science 14 Other Biology 3 Physical Science 3 Non-Science 6 Missing 1
State of Undergraduate Degree (*n*, % SA)	19, 48.7%
Year Undergraduate Degree Completed (Mdn, Range)	2009 (1998–2012)
Year Admitted into MD^b^	1 × 2009, 2 × 2010, 3 × 2011, 5 × 2012, 28 × 2013

^a^All were selected using the same criteria so combined for analysis

^b^Only one person had an intermitting year between their medical qualification and internship

### Baseline selection and assessment data (see Table 3)

Interns had a mean GAMSAT total score of 63.7 (SD = 4.9), and interview mean score of 74.8 (SD = 15.0). The mean wGPA was 87.7 (SD = 11.8), which equates to 6.14 using a 7-point GPA scale (Australian) or 3.51 on a 4-point scale (North American). The overall mean rank score, a composite of GAMSAT, wGPA and Interview score, was 226.2 (SD = 19.4). Seven of the 39 interns (17.9%) required at least one Improving Performance Action Plan across their internship (range 1–7).

**Table 3:** Description of Selection Scores and Workplace Outcomes (*n* = 39)

	Mean (SD)
SELECTION SCORES	
GAMSAT	
Section 1 (Reasoning in the Humanities and Social Sciences)	62.7 (5.7)
Section 2 (Written Communication)	65.3 (6.9)
Section 3 (Reasoning in the Biological Sciences)	63.4 (7.8)
Total	63.7 (4.9)
wGPA %	87.7 (11.8)
Interview	
Communication (/15)	11.1 (2.6)
Motivation (/15)	11.5 (2.8)
Decision Making (/15)	10.9 (2.4)
Learning Style (/15)	11.3 (2.5)
Prosocial Attitudes (/15)	11.0 (3.1)
Personal Management (/15)	11.6 (2.4)
Global (/15)^a^	11.4 (2.7)
Total (%)	74.8 (15.0)
Rank (GAMSAT Total + wGPA + Interview %)	226.2 (19.4)
INTERN ASSESSMENTS	
Domain 1 (Science and Scholarship)	3.8 (0.4)
Domain 2 (Clinical Practice)	3.9 (0.4)
Domain 4 (Professionalism and Leadership)	4.0 (0.4)
Total	3.9 (0.4)
No. of IPAPs (Improving Performance and Action Plans required)	0.5 (1.4)
Global^b^ (Single assessment of unsatisfactory, borderline or satisfactory)	2.9 (0.2)

^a^This reflects an interviewer’s overall assessment of an applicant’s suitability for the medical profession and may take into account any aspect of an applicant’s performance during the interview

^b^This is a workplace supervisor’s overall assessment of an intern’s workplace performance across their internship term

### Exploratory analysis

#### Sociodemographic data (see Table 4)

Age and years since entry into the medical course had a number of associations of at least *r* = .20. Being older was associated with poorer scores for all outcomes except the number of IPAPs, with small to moderate negative correlations ranging from *r* = −.27 for global performance to *r* = −.38 for Domain 2 (Clinical Practice). The greater the number of years between being first admitted into the course and undertaking internship, the poorer the performance across all outcomes, with moderate to high negative correlations ranging from *r* = −.56 for Domain 4 (Professionalism and Leadership) and *r =* −.79 for global performance and positive association with number of Improving Performance Action Plans (*r* = .71). There was a small positive association of *r* = .20 between the level of science in background degrees and performance on Domains 2 (Clinical Practice) and 4 (Professionalism and Leadership).

**Table 4:** Correlations (Pearson’s *r*) between sociodemographic characteristics and assessments (*n* = 39)

	Assessments					
	Domain 1	Domain 2	Domain 4	Total	No. of IPAPs	Global
	Science and Scholarship	Clinical Practice	Professionalism and Leadership	Domains 1 + 2 + 4	Improving Performance Action Plan Required	Overall Performance
Age	−.37*	−.38*	−.26	−.35*	.16	−.27
Gender	−.03	−.03	−.01	−.03	−.08	.08
Years^a^	−.65**	−.58**	−.56**	−.63**	.71**	−.79**
Rural	.07	.05	.09	.08	−.05	.02
Science background^b^	.06	.20	.20	.16	−.11	.09

*p < .05, **p < .01

^a^Number of years between entry into the medical course and internship year (only one intern had a year off in between so this equals length of time taken to complete medical course)

^b^Undergraduate degree coded for ‘science’ focus using Craig et al. [19]

#### Selection and outcome data (see Table 5)

Overall selection rank had moderate correlations with all assessments. A higher selection rank was associated with better performance with moderate associations ranging from *r* = .36 for Domain 1 (Science and Scholarship) to *r* = .48 for Domain 4 (Professionalism and Leadership). Selection rank was negatively correlated with number of Improving Performance Action Plans (*r* = −.50).

**Table 5:** Correlations (Pearson’s *r*) between selection criteria and assessments (*n* = 39)

	Assessments					
	Domain 1	Domain 2	Domain 4	Total	No. of IPAPs	Global
	Science and Scholarship	Clinical Practice	Professionalism and Leadership	Domains 1 + 2 + 4	Improving Performance Action Plan required	Overall Performance
Rank	.36*	.46**	.48**	.45**	−.50**	.45**
GAMSAT						
Section 1 (Reasoning in Humanities and Social Sciences)	.14	.13	.17	.16	−.17	.15
Section 2 (Written Communication)	−.11	−.04	.03	−.04	−.06	−.05
Section 3 (Reasoning in the Biological Sciences)	.23	.10	.17	.17	−.12	.19
Total	.17	.09	.18	.15	−.15	.17
wGPA	.41*	.37*	.33*	.39*	−.29	.34*
Interview Total	.09	.27	.30	.23	−.37*	.27
Communication	−.04	.14	.20	.10	−.25	.16
Motivation	−.05	.15	.19	.10	−.24	.19
Decision Making	−.04	.13	.18	.09	−.19	.09
Learning Style	.32	.42*	.46**	.42*	−.49**	.34*
Prosocial Attitudes	.09	.29	.31	.24	−.37*	.25
Personal Management	.20	.33	.31	.29	−.42*	.35*
Global	.17	.36*	.37*	.31	−.43**	.36*

*p < .05, **p < .01

Rank, as described above, comprises three individual selection scores: GAMSAT, wGPA and Interview scores. With respect to GAMSAT, results were unremarkable except for one small positive correlation of *r* = .23 between GAMSAT Section 3 (Reasoning in Biological Sciences) and Domain 1 (Science and Scholarship). wGPA was moderately correlated with all domain assessments, ranging from *r* = .33 for Domain 4 (Professionalism and Leadership) and *r* = .41 for Domain 1 (Science and Scholarship). It was negatively correlated with number of Improving Performance Action Plans (*r* = −.29).

There were small to moderate correlations between the overall interview score and all assessments (except for Domain 1, Science and Scholarship), ranging from *r* = .23 for total performance to *r* = .30 for Domain 4 (Professionalism and Leadership). A higher interview score was negatively correlated with number of Improving Performance Action Plans (*r* = −.37). When the seven subscales of the Interview were examined, learning style, prosocial attitudes, personal management and the global score were associated with almost all assessments. These ranged from *r* = .24 between prosocial attitudes and Total Score to *r* = −.49 between learning style and number of Improving Performance Action Plans.

## Discussion

Medical student selection scores were associated with internship performance using multiple assessments. Selection rank explained 25% of variance in the number of times interns required individualised supportive plans to improve their performance. When looking at the component scores of selection rank, both wGPA and the Interview had multiple and generally moderate positive associations with all performance measures. GAMSAT had a single relationship with performance, with Section 3 (Reasoning in the Biological Sciences) having a small association with Domain 1 (Science and Scholarship).

The findings regarding the Interview are interesting. Its associations with Domain 1 (Science and Scholarship) are generally small, which would be expected given it does not seek to assess these outcomes. However, amongst the multiple associations identified, the Interview domain of “Learning Style” had consistently higher correlations with all other outcomes. Given the internship year is considered possibly the most important postgraduate year for learning [21], this may suggest good construct validity for this Interview domain in particular. Some may be surprised about the demonstrated relationships between a panel interview and workplace outcomes, given ‘traditional interviews’ have been reported as lacking validity. Yet it is the degree of structure within any interview that contributes to its reliability and validity [6], and this particular panel interview is highly structured.

Both age and number of years between the entry year into the medical course and internship were also associated with workplace performance. They are intrinsically related to each other (the longer a student takes to complete the course then the older they will be), but how they relate to other possible predictor variables of performance is likely complex and could not be examined with such a small sample size and bivariate analyses. Only simple correlations rather than regressions were possible with the available data. However, the associations between number of years in the course and poorer performance were strong (up to *r* = .79), and given others have reported that being older is associated with poorer in-course performance [22] and poorer junior doctor workplace performance [8], this warrants further examination. It may be that age intercorrelates with other factors such as increasing family commitments (e.g., children) or other caring responsibilities. Understanding these factors may facilitate better support of interns.

An earlier retrospective cohort study at Flinders University found that wGPA was the most robust predictor of all 4 years of in-course performance, GAMSAT was predictive in the two pre-clinical years and the interview was predictive of performance in the final two clinical years [23]. Whilst the relative contributions of these selection scores to junior doctor workplace performance cannot be identified in the current study due to the use of simple correlations, findings are sympathetic with same. It appears that GPA and the Interview continue to be important, and GAMSAT Section 3 (Reasoning in the Biological Sciences) re-emerges in the internship year.

Overall the findings reveal that the selection scores used to select graduate entry medical students into this medical course were related to postgraduate junior doctor workplace performance. This is despite (1) a truncated range of selection scores (those with lower scores are “selected out” before medical school), (2) internship being at least 4 to 5 years distal to student selection, and (3) substantial heterogeneity in the outcome data (both in terms of different assessors and clinical rotations). The positive findings may reflect the methodological strength of this study. The use of data matching protected the results from possible self-selection bias as all Flinders University graduates who worked as interns at SALHN were included, equating to a 100% response rate.

As an exploratory study using correlations, findings carry the risk of Type 1 errors and causality cannot be implied. The use of bivariate statistics means that potential predictor variables may be intercorrelated and it is not possible to identify the predictive value of any particular set of variables. It is also not possible to directly extrapolate findings to other jurisdictions of practice or universities. Nevertheless, this study contributes to the broader scant international research concerning selection in relation to performance. In particular it identifies potential relationships of interest for GPA, Interview and GAMSAT Section 3 (Reasoning in the Biological Sciences) with junior doctor workplace performance. This work extends previous Australian research [8] by being the only known study to examine these relationships in a graduate entry medical school cohort rather than undergraduate cohort.

It seems appropriate to recommend that longitudinal research is now required, following graduate cohorts across the medical education continuum at multiple institutions. However, the variations in selection practices both within and between countries make it practically difficult to do this even using in-course outcomes, let alone with more distal outcomes. In the absence of a “single undisputed gold standard that measures the performance of a practicing health professional” [5] (p1094) it will be a challenge to undertake predictive validity studies of outcomes such as workplace performance.

Arguably what should come first are collaborative projects involving multiple medical schools and medical education providers that map a range of desired outcomes across a medical career trajectory (spanning medical school to independent practice) to selection policies and practice. We agree with the most recent Ottawa Consensus Statement which recommends the “use of validated taxonomies of desirable behaviors that indicate success as a healthcare practitioner to judge the quality of selection, which are contextualised and relevant across stages of training” [5] (p1098). First, however, such indicators of success must be identified and agreed upon. Only then does it make sense to undertake longitudinal predictive validity studies. In the meantime, smaller proof of concept studies demonstrating that selection scores can be associated with junior doctor workplace performance will provide further insight into the nature of this relationship. Given that slower progression through the course was also associated with poorer performance, further research should examine this phenomenon.

## Conclusions

Selection tools for entry into medicine are the most critical assessments given most medical students end up in clinical practice. Multiple elements of student selection scores had small to moderate associations with junior doctor workplace performance in internship year, with up to 25% of variance explained depending on the outcome variable. Both prior academic performance from an undergraduate degree (GPA) and panel interview scores across multiple domains appeared to be most important, although a rank comprised of multiple scores was also associated with outcomes. As extant selection research focusses predominantly on in-course outcomes, and those that do extend to post graduate outcomes focus on demonstration of competency as opposed to day to day practice, this study contributes to a significant gap in what is known. Future collaborative research should map desired outcomes across the medical education trajectory to selection and explore the impact of changes to selection which focus on assessment of these domains. The phenomenon of slower course progression being associated with poorer workplace performance should also be examined.

## Data Availability

The datasets generated and/or analysed during the current study are not publicly available as they contain highly personal confidential data and institutional workplace data not approved for release external to the University or health service. Data are required to be held solely in the University’s own confidential digital environment.

## Abbreviations

GAMSAT: Graduate Australian Medical School Admissions Test
INTERNSHIP: The intern year is a transition to practice year, also referred to as Postgraduate Year 1 (PGY1), which must be completed in order for a graduate to receive general registration as a medical practitioner in Australia. It comprises 47 weeks of supervised clinical experience in various settings, at the end of which the employing health agency must certify satisfactory completion of required rotations
IPAP: Improving Performance Action Plan
SALHN: Southern Adelaide Local Health Network
wGPA: Weighted Grade Point Average from a prior undergraduate Internship, where (GPA Year 1 × 1) + (GPA Year 2 × 2) = (GPA Year 3 × 3) / 6
